# Differences in biomarker testing in non-small cell lung cancer: real-world outcomes within an integrated healthcare system

**DOI:** 10.3389/fsurg.2025.1632360

**Published:** 2025-11-06

**Authors:** William P. Carroway, Nathan J. Alcasid, Alberto Jarrin Lopez, Kenneth Williams, Varada Sarovar, Huyun Dong, Wendy Dyer, Jingrong Yang, Lori C. Sakoda, Jeffrey B. Velotta

**Affiliations:** 1 Department of Surgery, University of California San Francisco East Bay, Oakland, CA, United States; 2 Division of Research, Kaiser Permanente Northern California, Pleasanton, CA, United States; 3 Division of Thoracic Surgery, Department of Surgery, Kaiser Permanente Northern California, Oakland, CA, USA; 4 Department of Surgery, University of California San Francisco School of Medicine, San Francisco, CA, United States; 5 Department of Health Systems Science, Kaiser Permanente Bernard J. Tyson School of Medicine, Pasadena, CA, United States; 6 Department of Clinical Sciences, Kaiser Permanente Bernard J. Tyson School of Medicine, Pasadena, CA, United States

**Keywords:** non-small cell lung cancer (NSCLC), biomarker testing, next-generation sequencing(NGS), survival outcomes, health disparities

## Abstract

**Introduction:**

While biomarker testing can guide lung cancer treatment, its real-world application in community practice remains underexplored. This study examines the prevalence, predictors, and outcomes of biomarker testing in non-small cell lung cancer (NSCLC).

**Methods:**

This retrospective cohort study included adults diagnosed with primary NSCLC from 2013 to 2020 within a large integrated healthcare system. We linked cancer registry and electronic health records to determine the prevalence of biomarker testing, including single-gene, multi-gene, and next-generation sequencing (NGS), overall and stratified by patient characteristics including age, gender, race/ethnicity, smoking status, and stage. Multivariable regression analyses were conducted to identify independent predictors of biomarker testing and evaluate associations between type of biomarker testing and 3-year all-cause mortality, overall and stratified by stage.

**Results:**

Among 8,267 NSCLC patients, 38.9% received biomarker testing. Testing prevalence increased with disease stage: I (6.9%), II (18.0%), III (34.8%), IV (71.1%). Testing was more prevalent in patients aged <65 years, of Asian race, and who never smoked, lived in less deprived neighborhoods, and had non-squamous tumors. Younger age, never smoking, Asian race, and stage IV disease were independent predictors of biomarker testing. NGS vs. no testing was associated with 13% decreases in 3-year all-cause mortality.

**Conclusions:**

Biomarker testing prevalence was higher in advanced stage NSCLC as expected, with decreased 3-year mortality in patients who received NGS testing. Our findings in a large real-world diverse population suggest that broader uptake of comprehensive biomarker testing across all stages of NSCLC is warranted for improved outcomes.

## Introduction

Lung cancer is the leading cause of cancer-related death in the world (1). Despite the high burden of lung cancer throughout the world, epidemiology differences exist, particularly in persons who have never smoked (2). In the United States (U.S), people who do not smoke make up nearly 15% of lung cancers as opposed to Asian countries, where one-third of lung cancers arise in people who do not smoke (2). Gender differences also exist where women may be at higher risk of lung cancer than men, with reported incidence rates per 100,000 person-years of 14%–21% in women and 5%–14% in men (3). However, current epidemiologic data is lacking as prior studies were limited to less than 180 cases without stratifying disease incidence and outcomes by race/ethnicity (2, 3). Thus, the epidemiology of lung cancer in people who do not smoke has not been adequately studied in the U.S. largely in part that most cancer registries do not collect information on smoking status, and few other data sources have an adequate number of people who do not smoke diagnosed with lung cancer (3–6). Another possible reason is that smoking is the predominant risk factor for lung cancer, which has overshadowed the importance of understanding lung cancer in persons who have never smoked.

The advent of biomarker testing has revolutionized the management of lung cancer. It has been well documented that there is increased prevalence of biomarker mutations, particularly epidermal growth factor receptor (EGFR), in Asian women who have never smoked with lung cancer (7, 8). Whether the potentially higher rate of biomarker mutations in Asian women who have never smoked is explained by genetics or environment is unclear (4). Nearly all lung tumors found in people who have never smoked are of non-small cell lung cancer (NSCLC) histology with a striking predominance of adenocarcinoma (AC) over squamous cell carcinoma (SCC) (4–6, 9). Additionally, targetable biomarkers such as EGFR, anaplastic lymphoma kinase (ALK), human epidermal growth factor 2 (HER2), and reactive oxidative species proto-oncogene 1 (ROS1) mutations are more frequently detected in the tumors of people who have never smoked (4, 5, 10). However, most studies have looked at a certain subset of lung cancer patients, mainly metastatic; thus, these studies do not represent the remaining 50% of NSCLC patients diagnosed with Stages I–III (4, 5, 10). Understanding biomarker prevalence and the associated outcomes across all stages of lung cancer related to various sociodemographic factors is a critical step in developing the evidence base needed to improve universal biomarker uptake.

Despite targeted therapy treatment improvements in NSCLC survival, comprehensive biomarker testing recommendations by the National Comprehensive Cancer Network (NCCN) continue to recommend complete next-generation sequencing (NGS) testing for advanced-stage disease only (11). Real-world studies have shown that biomarker testing penetration remains incomplete, particularly in community-based settings serving diverse, broadly representative patient populations (13–15). Substantial physician variation in test ordering exists and has been associated with differences in clinical outcomes (15), while testing rates and use of targeted therapy vary by practice type, demographic characteristics, and insurance status, revealing persistent inequities in access (13, 14). These disparities are especially significant as some mutations—such as EGFR in Asian women who do not smoke and ALK in younger patients with adenocarcinoma—are more common in specific sociodemographic and clinical cohorts (11–14).

There remains a significant knowledge gap regarding biomarker testing prevalence, determinants, and outcomes across all NSCLC stages in real-world, diverse populations. Understanding such disparities, particularly those related to race/ethnicity, age, and smoking status is essential for addressing equity in lung cancer care. The primary objective of this study was to examine the prevalence, sociodemographic and clinical factors, and mortality outcomes associated with biomarker testing, including next generation sequencing (NGS) and non-NGS modalities, among patients with NSCLC of all stages within a racially and socioeconomically diverse integrated healthcare system.

## Materials and methods

### Study design and population

This retrospective cohort study included adults ages 18–89 years diagnosed with primary NSCLC of any stage from 2013 to 2020 in an integrated healthcare system serving the Greater Bay Area and Central Valley regions of Northern California. This system currently provides comprehensive medical care services to 4.6 million enrolled health plan members—nearly 40% of the insured population in its service area—at 21 hospitals and over 240 outpatient clinics. Its population of health plan enrollees is relatively stable, sociodemographically diverse, and broadly representative of the residing population in Northern California. The integrated care setting enables robust examination of the cancer care continuum from diagnosis to survivorship. Our institutional review board approved a waiver of informed consent for this research.

The eligible study population was identified by linking individual-level cancer registry and electronic health records (EHR) described below. Patients diagnosed with a first primary NSCLC at ages 90 and older, prior lung cancer, or recurrent lung cancer were excluded. Patients followed for less than 90 days after NSCLC diagnosis were further excluded to minimize selection bias. Those with less than 90 days of follow-up, either due to death or health plan disenrollment, were presumably less likely to have had the opportunity to undergo and benefit from biomarker testing.

### Data sources and variables

Existing data from institutional EHR databases and registries were extracted for analysis. In addition to the integrated data systems that support care delivery and clinical operations, our institution maintains several registries for regulatory and research purposes. That includes a cancer registry, which captures information on all patients diagnosed or treated with cancer at affiliated facilities in accordance with national cancer registry data standards, and a vital statistics registry, which aggregates mortality data on all health plan enrollees from internal records, California state and U.S. Social Security Administration death files, and the National Death Index. Using EHR databases, receipt of biomarker testing and results following NSCLC diagnosis was determined for all patients. Biomarker testing encompassed non-NGS testing [i.e., by immunohistochemistry (IHC), fluorescence *in situ* hybridization (FISH), polymerase chain reaction (PCR)] and NGS testing. Despite the exclusion criterion on length of follow-up, patients were classified as being tested, even if biomarker testing occurred within 90 days of NSCLC diagnosis.

The following covariates were measured as of the date of NSCLC diagnosis to further describe and characterize the study population and subpopulations of interest in relation to biomarker testing: age, sex, race/ethnicity, neighborhood deprivation index (NDI), and clinical characteristics, such as smoking status, tumor stage, and histologic subtype. The NDI is a composite measure that reflects socioeconomic disadvantage within a neighborhood, incorporating factors such as income, education, employment, and housing quality, and serves as a proxy for patients’ social determinants of health (16). Smoking status was ascertained electronically, and if missing, by review of clinical notes to the extent possible. These covariates were selected because they could be readily obtained from institutional data sources and were posited to be associated with receipt of biomarker testing and outcomes.

### Statistical analyses

The prevalence of biomarker testing was calculated as the proportion of patients who underwent biomarker testing, irrespective of whether valid test results were returned. Prevalence was examined in the study population, overall and within selected subpopulations defined by sociodemographic and clinical factors.

All patients with missing covariate data, except on smoking status, were retained in analyses. Specifically, patients with missing data on race and ethnicity were combined with those of other or multiple race, while patients with missing data on NDI and tumor stage were classified separately into an “unknown” category. The few patients with missing data on smoking status were excluded, since none had received biomarker testing.

Logistic regression was used to estimate crude and adjusted odds ratios (OR) and 95% confidence intervals (CI) to identify sociodemographic and clinical factors associated with receipt of biomarker testing. Analyses were conducted overall and stratified by stage (I-III vs. IV). Since clinical guidelines have historically recommended biomarker testing for stage IV (metastatic) NSCLC, stratified analyses were performed to explore whether factors associated with biomarker testing differ by disease stage. Follow-up for 3-year all-cause mortality was measured from 90 days after NSCLC diagnosis (time zero) until the date of death, health plan disenrollment, or interval end (1,095 days), whichever occurred earliest. This specific endpoint was chosen since existing vital status registry data permitted at least 3 years of follow-up on all patients.

Among patients who had valid biomarker test results, crude and adjusted hazard ratios (HRs) and 95% confidence intervals (CIs) were calculated using Cox proportional hazards regression to evaluate the association between biomarker testing type (NGS vs. no testing and non-NGS testing vs. no testing) and mortality outcomes. These analyses were restricted to patients who had valid test results, as invalid test results would not inform appropriate treatment decisions and thereby have a potentially negative prognostic influence. Multivariable models were adjusted for potential confounders determined *a priori*, including age, sex, race/ethnicity, smoking status, NDI, tumor stage, and histologic subtype. Analyses were again conducted overall and stratified by stage. Model assumptions of proportional hazards were statistically tested and satisfied. All statistical analyses were performed using SAS version 9.4 (Cary, NC).

## Results

### Characteristics of the study population

The study cohort included 8,267 patients diagnosed with incident NSCLC between 2013 and 2020, with at least 90 days of follow-up after diagnosis. The population was diverse, representing a range of sociodemographic characteristics in terms of age, race/ethnicity, socioeconomic status, and smoking history (Table 1).

**Table 1:** Baseline demographic and clinical characteristics associated with biomarker testing Status in patients with primary NSCLC.

Characteristics	Overall (*n* = 8,267)	Tested (*n* = 3,216)	Not tested (*n* = 5,051)	Crude OR (95% CI)	Adjusted OR (95% CI)^a^
Age at diagnosis, *n* (%)					
18–54	502 (6.1)	310 (9.6)	192 (3.8)	3.2 (2.7–3.9)	1.9 (1.5–2.5)
55–64	1,531 (18.5)	698 (21.7)	833 (16.5)	1.7 (1.5–1.9)	1.3 (1.1–1.6)
65–74	2,939 (35.6)	1,113 (34.6)	1,826 (36.1)	1.2 (1.1–1.4)	1.1 (0.99–1.3)
75–89	3,295 (39.9)	1,095 (34.1)	2,200 (43.6)	1.0 (reference)	1.0 (reference)
Mean (SD)	70.5 (10.2)	68.6 (11.1)	71.7 (9.4)	–	–
Sex, *n* (%)					
Male	3,693 (44.7)	1,418 (44.1)	2,275 (45.0)	1.0 (reference)	1.0 (reference)
Female	4,574 (55.3)	1,798 (55.9)	2,776 (55.0)	1.0 (0.95–1.1)	1.0 (0.93–1.2)
Race and ethnicity, *n* (%)					
Asian	1,433 (17.3)	743 (23.1)	690 (13.7)	2.0 (1.8–2.2)	1.5 (1.2–1.7)
Black	679 (8.2)	268 (8.3)	411 (8.1)	1.2 (1.0–1.4)	1.2 (0.97–1.5)
Hispanic	648 (7.8)	249 (7.7)	399 (7.9)	1.1 (0.97–1.4)	0.98 (0.79–1.2)
White	5,146 (62.3)	1,815 (56.4)	3,331 (65.9)	1.0 (reference)	1.0 (reference)
Other, Multiple, or Unknown	361 (4.4)	141 (4.4)	220 (4.4)	1.2 (0.95–1.5)	1.2 (0.91–1.6)
Smoking status, *n* (%)^b^					
Current	1,583 (19.2)	520 (16.2)	1,063 (21.1)	0.90 (0.80–1.0)	0.85 (0.73–0.99)
Former	4,585 (55.5)	1,615 (50.2)	2,970 (58.8)	1.0 (reference)	1.0 (reference)
Never	2,095 (25.3)	1,081 (33.6)	1,014 (20.1)	2.0 (1.8–2.2)	1.3 (1.1–1.5)
Neighborhood deprivation index, *n* (%)					
Quartile 1 (least deprived)	2,118 (25.6)	910 (28.3)	1,208 (23.9)	1.4 (1.2–1.6)	1.5 (1.3–1.8)
Quartile 2	2,102 (25.4)	820 (25.5)	1,282 (25.4)	1.2 (1.0–1.3)	1.2 (1.0–1.4)
Quartile 3	2,054 (24.9)	774 (24.1)	1,280 (25.3)	1.1 (0.96–1.2)	1.1 (0.94–1.3)
Quartile 4 (most deprived)	1,982 (24.0)	705 (21.9)	1,277 (25.3)	1.0 (reference)	1.0 (reference)
Stage at diagnosis, *n* (%)					
I	2,571 (31.1)	177 (5.5)	2,394 (47.4)	1.0 (reference)	1.0 (reference)
II	756 (9.1)	136 (4.2)	620 (12.3)	3.0 (2.3–3.8)	3.5 (2.8–4.5)
III	1,555 (18.8)	541 (16.8)	1,014 (20.1)	7.2 (6.0–8.7)	10.1 (8.3–12.2)
IV	3,252 (39.3)	2,313 (71.9)	939 (18.6)	33.3 (28.1–39.5)	37.6 (31.5–44.9)
Unknown	133 (1.6)	49 (1.5)	84 (1.7)	8.1 (5.4–11.9)	11.0 (7.2–16.7)
NSCLC histologic subtype					
Adenocarcinoma	6,085 (73.6)	2,728 (84.8)	3,357 (66.5)	4.7 (4.0–5.4)	4.8 (4.0–5.6)
Squamous cell carcinoma	1,643 (19.9)	244 (7.6)	1,399 (27.7)	1.0 (reference)	1.0 (reference)
Other	539 (19.9)	244 (7.6)	295 (5.8)	4.7 (3.8–5.9)	3.8 (2.9–4.9)

a Fully adjusted model including all listed variables.

b Excludes 4 patients with missing data, all of whom did not receive biomarker testing.

### Prevalence of biomarker testing

**Figure 1:**
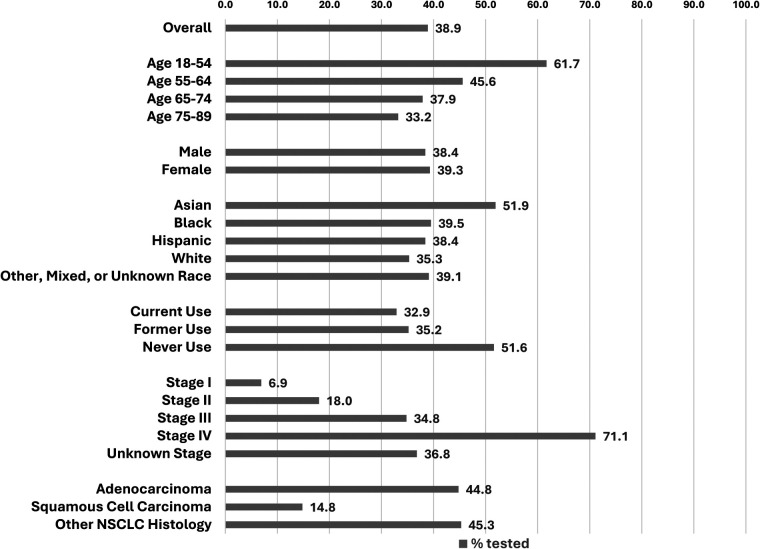
Prevalence of biomarker testing, overall and by selected demographic and clinical factors.

Overall, 38.9% (3,216 patients) received biomarker testing (Figure 1). Receipt of biomarker testing decreased by age at NSCLC diagnosis, with the highest prevalence observed in patients aged 18–54 (61.7%). The prevalence of biomarker testing in Asian, Black, Hispanic, and White populations was 51.9%, 39.5%, 38.4%, and 35.5%, respectively. By smoking status, biomarker testing was most prevalent among those who never smoked (51.6%), compared to those who formerly smoked (35.2%) and actively smoked (32.9%) at NSCLC diagnosis. Biomarker testing increased with higher stage, specifically 6.9% for stage I, 18.0% for stage II, 34.8% for stage III, and 71.1% for stage IV. By histology, biomarker testing was much lower in those with squamous cell carcinoma (14.8%) than patients with adenocarcinoma (44.8%) or other NSCLC histology (45.3%).

### Sociodemographic and clinical factors associated with biomarker testing

Crude and multivariable logistic regression analyses showed relatively consistent associations between sociodemographic and clinical factors and receipt of biomarker testing (Table 1). All factors examined, except for sex, were independently associated with biomarker testing receipt. In multivariable analysis, higher odds of testing were found for both age groups under 65 (vs. 65–74) [for 18–54: adjusted OR 1.9; 95% CI: 1.5–2.5; for 55–64: adjusted OR 1.3; 95% CI: 1.1–1.6]; Asian (vs. White) race [adjusted OR 1.5; 95% CI: 1.2–1.7]; lowest (vs. highest) quartile of NDI [adjusted OR: 1.5 95% CI: 1.3–1.8]; stages II–IV (vs. I) [for stage II: adjusted OR 3.5; 95% CI: 2.8–4.5; for stage III: adjusted OR 10.1 95% CI: 8.3–12.2; for stage IV: adjusted OR 37.6; 95% CI: 31.5–44.9]; and adenocarcinoma and other NSCLC (vs. squamous cell carcinoma) histology [for adenocarcinoma: adjusted OR 4.8; 95% CI: 4.0–5.6; for other NSCLC: adjusted OR 3.8; 95% CI: 2.9–4.9]. For smoking status, odds of testing were higher for never smoking [adjusted OR 1.3; 95% CI: 1.1–1.5] but lower for current smoking [adjusted OR 0.85; 95% CI: 0.73–0.99] relative to former smoking.

Similar association patterns were observed in analyses stratified by stage. However, the associations observed, especially for age and histologic subtype, were stronger in magnitude among patients with stage IV NSCLC (Table 2). Additionally in this stage-specific subgroup, a higher odds of testing was noted for Black (vs. White) race [adjusted OR 1.4; 95% CI: 1.0–1.9].

**Table 2:** Baseline demographic and clinical characteristics associated with biomarker testing status in patients with primary NSCLC by stage.

Characteristics	Stage I–III NSCLC (*n* = 4,882)				Stage IV NSCLC (*n* = 3,252)			
	Tested (*n* = 854)	Not tested (*n* = 4,028)	Crude OR (95% CI)	Adjusted OR (95% CI)^a^	Tested (*n* = 2,313)	Not tested (*n* = 939)	Crude OR (95% CI)	Adjusted OR (95% CI)^a^
Age at diagnosis, *n* (%)								
18–54	61 (7.1)	143 (3.6)	2.1 (1.5–2.8)	1.6 (1.1–2.3)	248 (10.7)	46 (4.9)	3.1 (2.2–4.3)	2.6 (1.8–3.7)
55–64	166 (19.4)	663 (16.5)	1.2 (0.99–1.5)	1.0 (0.81–1.3)	524 (22.6)	164 (17.5)	1.8 (1.5–2.3)	1.8 (1.4–2.3)
65–74	266 (31.2)	1,474 (36.6)	0.87 (0.74–1.0)	0.86 (0.71–1.0)	832 (36.0)	321 (34.2)	1.5 (1.2–1.8)	1.5 (1.3–1.8)
75–89	361 (42.3)	1,748 (43.4)	1.0 (reference)	1.0 (reference)	709 (30.7)	408 (43.4)	1.0 (reference)	1.0 (reference)
Mean (SD)			–	–			–	–
Sex, *n* (%)								
Male	353 (41.3)	1,746 (43.4)	1.0 (reference)	1.0 (reference)	1,041 (45.0)	480 (51.1)	1.0 (reference)	1.0 (reference)
Female	501 (58.7)	2,282 (56.6)	1.1 (0.93–1.3)	1.0 (0.87–1.2)	1,272 (55.0)	459 (48.9)	1.3 (1.1–1.5)	1.1 (0.89–1.2)
Race and ethnicity, *n* (%)								
Asian	187 (21.9)	550 (13.7)	1.8 (1.5–2.2)	1.5 (1.2–1.9)	547 (23.7)	130 (13.8)	2.0 (1.6–2.5)	1.4 (1.1–1.8)
Black	72 (8.4)	337 (8.4)	1.1 (0.88–1.5)	1.1 (0.79–1.5)	193 (8.3)	67 (7.1)	1.4 (1.0–1.9)	1.4 (1.0–1.9)
Hispanic	58 (6.8)	311 (7.7)	1.0 (0.75–1.3)	0.87 (0.63–1.2)	186 (8.0)	77 (8.2)	1.2 (0.88–1.5)	1.1 (0.82–1.5)
White	494 (57.9)	2,656 (65.9)	1.0 (reference)	1.0 (reference)	1,292 (55.9)	623 (66.4)	1.0 (reference)	1.0 (reference)
Other, Multiple, or Unknown	43 (5.0)	174 (4.3)	1.3 (0.94–1.9)	1.4 (0.96–2.1)	95 (4.1)	42 (4.5)	1.1 (0.75–1.6)	0.95 (0.63–1.4)
Smoking status, *n* (%)^b^								
Current	144 (16.9)	834 (20.7)	0.88 (0.72–1.1)	0.90 (0.72–1.1)	363 (15.7)	206 (21.9)	0.85 (0.70–1.0)	0.77 (0.61–0.96)
Former	465 (54.4)	2,376 (59.0)	1.0 (reference)	1.0 (reference)	1,128 (48.8)	543 (57.8)	1.0 (reference)	1.0 (reference)
Never	245 (28.7)	817 (20.3)	1.5 (1.3–1.8)	1.3 (1.0–1.6)	822 (35.5)	189 (20.1)	2.1 (1.7–2.5)	1.3 (1.0–1.6)
Neighborhood deprivation index, *n* (%)								
Quartile 1 (least deprived)	237 (27.8)	1,014 (25.2)	1.3 (1.1–1.6)	1.3 (1.0–1.7)	661 (28.6)	179 (19.1)	1.9 (1.5–2.3)	1.8 (1.4–2.3)
Quartile 2	249 (29.2)	1,023 (25.4)	1.4 (1.1–1.7)	1.3 (1.0–1.6)	563 (24.3)	235 (25.0)	1.2 (0.98–1.5)	1.1 (0.86–1.4)
Quartile 3	189 (22.1)	998 (24.8)	1.1 (0.85–1.3)	1.0 (0.81–1.3)	566 (24.5)	261 (27.8)	1.1 (0.89–1.3)	1.2 (0.93–1.5)
Quartile 4 (most deprived)	177 (20.7)	991 (24.6)	1.0 (reference)	1.0 (reference)	518 (22.4)	262 (27.9)	1.0 (reference)	1.0 (reference)
Stage at diagnosis, *n* (%)								
I	177 (20.7)	2,394 (59.4)	1.0 (reference)	1.0 (reference)	–	–	–	–
II	136 (15.9)	620 (15.4)	3.0 (2.3–3.8)	3.4 (2.7–4.4)	–	–	–	–
III	541 (63.4)	1,014 (25.2)	7.2 (6.0–8.7)	9.4 (7.8–11.5)	–	–	–	–
IV	–	–	–	–	2,313 (90.8)	939 (74.5)	–	–
NSCLC histologic subtype								
Adenocarcinoma	690 (80.8)	2,753 (68.3)	2.4 (2.0–3.0)	3.4 (2.7–4.3)	2,019 (87.3)	583 (62.1)	7.6 (6.1–9.6)	6.6 (5.3–8.4)
Squamous cell carcinoma	112 (13.1)	1,091 (27.1)	1.0 (reference)	1.0 (reference)	131 (5.7)	288 (30.7)	1.0 (reference)	1.0 (reference)
Other	52 (6.1)	184 (4.6)	2.7 (1.9–4.0)	2.8 (1.9–4.1)	163 (7.0)	68 (7.2)	5.3 (3.7–7.5)	4.9 (3.4–7.0)

a Fully adjusted model including all listed variables.

b Excludes 2 patients with missing data who did not receive biomarker testing.

### All-cause mortality associated with biomarker testing

As expected, observed associations differed between crude and multivariable Cox regression analyses, since mortality is inherently associated with tumor stage. As shown in Table 3, patients who underwent NGS testing had a lower mortality risk at 3 years (adjusted HR 0.87; 95% CI: 0.77–0.97) after NSCLC diagnosis, while patients who underwent non-NGS testing had a higher mortality at 3 years (adjusted HR 1.3; 95% CI: 1.2–1.4) after NSCLC diagnosis, compared to those with no testing. In multivariable analyses stratified by sage, the association of NGS testing with decreased mortality appeared more pronounced and specific to patients with stage IV NSCLC (adjusted HR 0.76; 95% CI: 0.61–0.87), and the association of non-NGS testing with increased mortality appeared more pronounced and specific to patients with stage I-III NSCLC (adjusted HR 1.8; 95% CI: 1.6–2.1).

**Table 3:** All-Cause mortality associated with biomarker test type in patients with primary NSCLC, overall and by stage.

	All patients^a^		
	Deaths (*n*)	Crude HR (95% CI)	Adjusted HR (95% CI)^b^
Biomarker test type			
No valid test	2,393	1.0 (reference)	1.0 (reference)
Non-NGS	1,494	2.2 (2.1–2.4)	1.3 (1.2–1.4)
NGS	382	1.5 (1.3–1.6)	0.87 (0.77–0.97)
	Patients with stage I–III NSCLC		
	Deaths (*n*)	Crude HR (95% CI)	Adjusted HR (95% CI)^b^
Biomarker test type			
No valid test	1,335	1.0 (reference)	1.0 (reference)
Non-NGS	308	2.2 (2.0–2.6)	1.8 (1.6–2.1)
NGS	76	1.3 (1.0–1.6)	1.0 (0.81–1.3)
	Patients with stage IV NSCLC		
	Deaths (*n*)	Crude HR (95% CI)	Adjusted HR (95% CI)^b^
Biomarker test type			
No valid test	1,004	1.0 (reference)	1.0 (reference)
Non-NGS	1,173	0.94 (0.86–1.0)	1.0 (0.96–1.1)
NGS	291	0.64 (0.56–0.73)	0.76 (0.61–0.87)

a Includes patients with NSCLC of unknown stage.

b Adjusted for age, sex, race and ethnicity, smoking status, neighborhood deprivation index, stage at diagnosis, and NSCLC histologic subtype.

## Discussion

This multicenter retrospective study represents one of the most comprehensive investigations into the prevalence and factors associated with biomarker testing in NSCLC within a real-world community practice setting. Our main findings reveal that 38.9% of NSCLC patients received biomarker testing during the study period, with higher rates observed for younger age at diagnosis, Asian race, never smoking status, stage IV disease, and non-squamous histology. Additionally, NGS testing was associated with lower mortality in patients with stage IV NSCLC, while non-NGS testing was associated with higher mortality in patients with stage I-III NSCLC. This study underscores that despite the great potential of biomarker testing in personalizing NSCLC treatment and improving outcomes, its utilization varies widely among different populations and cancer stages.

At first glance, our finding of 38.9% of NSCLC patients receiving biomarker testing may seem lower than other recent studies which revealed 89.3% (12), 68.7% (17), 79.2% (18) biomarker testing rates. However, it is important to point out that we included all stages of NSCLC, whereas other studies published on biomarker testing uptake focused on advanced/metastatic NSCLC (5, 12). Thus, to our knowledge this is one of the largest studies to capture overall biomarker prevalence rates for all stages of NSCLC taking into account various sociodemographic factors. As our study period of NSCLC diagnosis spanned between January 2013 and December 2020, the higher prevalence of biomarker testing observed for stage IV disease (71.1%) is consistent with NCCN recommendations from this time period, as updated biomarker testing recommendations in NSCLC stages IB to IIIA had not yet been updated until September 2020 (19).

Our findings align with previous research showing that certain sociodemographic factors, such as younger age, Asian race, and never-smoking status, are associated with higher rates of biomarker testing in NSCLC (20–22). In our study, biomarker testing was performed among 35.3% of White patients and 39.5% of Black patients. This suggests non-Asian racial/ethnic equity in testing (all types) across our specific health system. This data is in alignment with prior findings which revealed 76.4% of White patients and 73.6% of Black patients underwent at least one single molecular test or NGS testing (*p* = 0.03) (23). It is also important to note that disparity in biomarker testing can exist on a more granular level. Although not the focus of our study, when comparing the differences in testing specifically for NGS and less established, non-targetable biomarkers, previous authors have described a statistically significant difference when comparing Black and White patients (22, 23). In our study, a striking difference in biomarker testing occurred when comparing Asian vs. all other racial/ethnic groups.

It is imperative to understand that studying differences in racial/ethnic background is paramount not only from a health equity standpoint, but also just as important to assess which populations may have more favorable responses to certain targeted therapies based on their specific associated biomarker alterations (24). Our study extends the existing literature by demonstrating that biomarker testing may be underutilized in early-stage disease and among older patients. These discrepancies with prior work may be attributed to differences in study populations and the evolving standards of care over time. The comprehensive nature of our dataset allows for a more nuanced understanding of these biomarker testing disparities within a real-world community setting.

Our findings of reduced one- and three-year mortality associated with comprehensive NGS testing in patients with stage IV NSCLC and of increased one- and three-year mortality associated with non-NGS testing in patients with stage I–III NSCLC is unique due to the fact that we included patients with all stages of NSCLC. Recent studies utilizing Flatiron Health EHR data showed similar results with improved overall survival in NSCLC patients who received biomarker testing in advanced and/or metastatic disease vs. those who did not (25). Although unexpected, the observed association of non-NGS testing with increased mortality after stage I-III NSCLC diagnosis may be explained by the lack of adjustment for receipt and type of initial treatment in our analyses, especially if non-NGS testing occurred preferentially in patients with unresectable or highly aggressive tumors. Several potential limitations to the validity of our findings were considered. Selection bias is a primary concern, given that patients who underwent biomarker testing differed from those who did not. We addressed this by employing multivariable Cox regression to control for various sociodemographic and clinical factors in examining mortality outcomes. As noted above, another limitation is that we did not adjust for initial treatment, which could influence outcomes and potentially bias the observed associations. In addition, measurement bias was minimized through the use of consistent and standardized testing protocols across our integrated healthcare system, and influential factors, such as stage and smoking status, were accounted for in our analysis, ensuring that the observed associations were robust. Prior literature has demonstrated a relationship between the likelihood of undergoing biomarker testing and year progression from 2016 to 2020. (19) We speculate that this is secondary to regulatory approval and increased adoption of the available therapies for patients with actionable biomarkers. This is consistent with our study period having a low overall testing prevalence (38.9%) but a relatively high testing prevalence in patients with stage IV NSCLC. Lastly, while our integrated healthcare network mitigates access to care and lack of health insurance coverage, there may be some unmeasured or unknown confounding factors that cannot be accounted for in our study. However, we believe residual confounding is likely minimal as we have a fully insured population with easy access within 30 miles of any medical center and/or clinic.

In summary, this study provides critical insights into the prevalence, determinants, and outcomes of biomarker testing uptake in NSCLC patients within a diverse, real-world setting. Our findings highlight biomarker testing uptake differences based on sociodemographic and clinical characteristics. Among patients with stage IV NSCLC, NGS testing was associated with lower mortality at 3 years after diagnosis. We believe these findings reiterate the importance of implementing comprehensive biomarker testing for all stages of NSCLC Future work should involve implementation of equitable and systematic biomarker testing across all populations.

## Data Availability

The raw data supporting the conclusions of this article will be made available by the authors, without undue reservation.
